# Advances in Cornea

**DOI:** 10.2174/1874364101812010130

**Published:** 2018-07-23

**Authors:** M. Vanathi

**Affiliations:** Cornea & Ocular Surface, Cataract & Refractive Services, R. P. Centre for Ophthalmic Sciences, All India Institute Of Medical Sciences, New Delhi 110029, India; Fax: 91-11-26588919; E-mail: mvanathi.rpc@gmail.com

Recent advances in corneal practice & research have largely been centered around interests concerning the endothelial keratoplasty, corneal biomechanics and collagen cross linking of the corneal stroma. This special issue of OPEN OPHTHALMOLOGY JOURNAL addresses these interests with an elaboration of the outcomes of endothelial keratoplasty procedures of Descemet's Membrane Endothelial Keratoplasty (DMEK) and Pre-Descemet’s Endothelial Keratoplasty (PDEK), a perspective on future potent therapies for Fuch's Endothelial Corneal Dystrophy (FECD), a comprehensive review on corneal biomechanics in LASIK and Small Incision Lenticule Extraction (SMILE) and on practical applications of collagen cross linking along with the outcomes of Lasik Xtra.

Since, the introduction of the concept of Descemet’s membrane endothelial keratoplasty by Melles *et al.* in 2002 [1], the rapid time to visual rehabilitation has made it the more preferred endothelial keratoplasty procedure. Several techniques have been described for harvesting the donor graft for DMEK procedure. Following a 9 mm trephination of the Descemet's Membrane (DM), peripheral scoring was facilitated by forceps, Sinskey hook or blade, and peeling being completed by a single or double non-toothed forceps, with the graft remaining immersed in the preservative media in the viewing chamber [2, 3]. The required size trephination of the DM was then done. The success rates of DMEK graft preparation vary with the tissue preparation in eye bank or by experienced surgeons [3-5]. Marking of the edge of the DM graft with orientation marks, the blue cannula sign, hand held slit bean, intra operative OCT aided surgery, ink marks on the DM side of the graft have all been elucidated to ensure the right orientation while positioning the DM graft within the eye [6-12]. Reported endothelial cell loss rates range from 25 to 53% over long term follow up and air injection rates varying from 13 – 82%, primary graft failure rate of 0.1 to 12.5%, graft rejection rates of 0.8 to 5.1% have also been noted in various studies on DMEK [13]. Graft detachments, secondary glaucoma and cystoids macular edema after DMEK are also a cause of concern. In this issue, Basak *et al.* have described the results of DMEK in their first 100 cases using surgeon prepared DMEK roll prepared by a simplified technique. The excellent results obtained seem to stem from the fact that the surgeon was already an experienced Descemet’s stripping endothelial keratoplasty surgeon, with capitalization on the knowledge gained over the reported results of the experiences of various other clinicians combined with standardization of technique.

PDEK is a novel endothelial keratoplasty technique in which the composite of pre-Descemet's stromal layer (Dua's layer) with DM and endothelium is transplanted subsequently to the removal of the recipient's DM [14]. The scrolling characteristics of the pre-descemetic layer have been attributed to influence tissue preparation, handling, and un-scrolling in the eye during the endothelial keratoplasty [15]. This technique has been described to enable the use of infant donor cornea for endothelial keratoplasty facilitating donor lenticule preparation, insertion of the donor graft, or air bubble management without any difficulty [16]. The continuous pressurized air infusion using an air pump-assisted technique has been described earlier to enable good graft positioning and adherence apart from also providing optimal tamponade to stop hemorrhage during the peripheral iridectomy, increasing surgical space and maintaining anterior chamber depth and anterior chamber depth [17]. In this issue, the use of continuous pressurized air infusion from the fluid air exchange system of a posterior vitrectomy machine for multiple key steps of PDEK surgery such as descemetorhexis, Peripheral Iridectomy (PI), graft floatation, graft centration, graft edge unfolding, graft un-wrinkling and graft adhesion is being evaluated by Jacob S in PDEK surgery.

For over a long period of time, endothelial transplantation seemed to be the only option for the treatment of Fuch’s endothelial corneal dystrophy. FECD has been categorized into three types (i) as early-onset FECD, (ii) as identified genetic loci, and (iii) disease without known inheritance [18]. Missense mutation of the COL8A2 gene on chromosome 1 p34.3-p32 has been identified in early onset FECD and genetic mutations in four genes (SLCA411, TCF8, LOXHD1, and AGBL1) were reported as causal factors as well [19-24]. The article by Okamura *et al.* explores the current strategies on the use of cultures corneal endothelial cells either as sheets for transplantation or by direct injection of cultured corneal endothelial cells in the anterior chamber as therapeutic interventions. The success of obtaining regeneration of the corneal endothelium by co-injecting cultured corneal endothelial cells along with ROCK inhibitor into anterior chamber in animal studies has prompted their team to further research in human eyes [25]. This review is a vivid elucidation of the future therapies for FECD.

With increasing popularity of SMILE procedure as a flapless and minimally invasive form of Laser Vision Correction (LVC) for the treatment of myopia and myopic astigmatism, the debate on its effects on corneal biomechanics in comparison to other LVC procedures is being widely investigated. Studies on the long-term outcomes of SMILE point towards its superior performance which shows excellent stability for higher myopia. SMILE also seems to have an advantage in terms of dry eye occurrence postoperatively over LASIK with better Schirmer's, tear film break up time, corneal sensitivity, and corneal nerve regeneration following SMILE as compared to LASIK [26]. The intact Bowman's Membrane (BM) and the corneal anterior stromal lamellae in SMILE procedure may perhaps be responsible for better corneal biomechanics over LASIK and PRK procedures. The flap-based LASIK and Refractive Lenticule Extraction (ReLEx) and flap-free ReLEx smile were noted by Pedersen *et al.* to result in a similar reduction in corneal biomechanics when evaluated by Corvis ST and ORA [27]. The decrease in corneal biomechanics was seen to be lesser in SMILE than with LASIK in myopia greater than -6.00 D [28]. Agca *et al.* found a decrease in Corneal Hysteresis (CH) and Corneal Resistance Factor (CRF) in SMILE and did not find differences in postoperative CH or CRF values between SMILE and femto-LASIK treatments [29]. However, considering that there seem to be conflicting reports on this in recent literature [30-34], the evidence on corneal biomechanics affection being less in SMILE is still inconclusive. In this issue, Iben *et al.* discuss the review of corneal biomechanical properties following LASIK and SMILE for myopia and myopic astigmatism.

This issue also covers a comprehensive review on current trends in practical applications of corneal collagen cross-linking which is a subject of interest to all in corneal practice in recent times. LASIK Xtra is a recently described technique which combines LASIK and accelerated CXL performed simultaneously in the same sitting. Lasik Xtra is thought to afford early stabilization of the cornea after LASIK, and enhance the predictability of refractive outcomes in highly myopic eyes [35-36]. Lim Li *et al.* also discuss their outcome of simultaneous accelerated corneal cross linking and laser *in situ* keratomileusis for the treatment of high myopia.
